# Pathogenicity and molecular characterization of a GI-19 infectious bronchitis virus isolated from East China

**DOI:** 10.3389/fvets.2024.1431172

**Published:** 2024-08-07

**Authors:** Qi Wu, Mengcheng Xu, Dengle Wei, Xuehua Zhang, Ding Li, Mei Mei

**Affiliations:** ^1^Institute of Veterinary Immunology and Engineering, Jiangsu Academy of Agricultural Sciences, Nanjing, China; ^2^GuoTai (Taizhou) Center of Technology Innovation for Veterinary Biologicals, Taizhou, China; ^3^Ministry of Education Key Laboratory for Avian Preventive Medicine, Yangzhou University, Yangzhou, Jiangsu, China; ^4^Jiangsu Key Laboratory of Food and Safety-State Key Laboratory Cultivation Base, Ministry of Science and Technology, Nanjing, China

**Keywords:** infectious bronchitis virus, phylogenetic analysis, pathogenicity, tissue tropism, GI-19 genotype

## Abstract

Infectious bronchitis virus (IBV) is responsible for avian infectious bronchitis, a disease prevalent in countries with intensive poultry farming practices. Given the presence of multiple genotypic strains in China, identifying the regionally dominant genotypes is crucial for the implementation of effective prevention and control measures. This study focuses on the IBV strain CK/CH/WJ/215, isolated from a diseased commercial chicken flock in China in 2021. The CK/CH/WJ/215 isolate was genetically characterized through complete S1 sequence analysis. Phylogenetic comparisons were made with prevalent vaccine strains (H120, LDT3-A, and 4/91). Glycosylation patterns in the S1 protein were also analyzed. Pathogenicity was assessed in 7-day-old specific-pathogen-free chicks, monitoring morbidity, mortality, and tissue tropisms. Phylogenetic analysis clustered the CK/CH/WJ/215 isolate within the GI-19 lineage. Identity with the vaccination strains H120, LDT3-A, and 4/91 was low (75.7%, 78.6%, and 77.5% respectively). Novel glycosylation sites at positions 138 and 530 were identified compared to H120 and LDT-A. The isolate demonstrated nephropathogenic characteristics, causing 100% morbidity and 73.3% mortality in SPF chicks, with broader tropisms in tissues including trachea, lungs, kidneys, and bursa of Fabricius. Comprehensive genetic and pathological investigations revealed significant differences between the CK/CH/WJ/215 isolate and common vaccine strains, including novel glycosylation sites and a strong multiorgan infective capability. These findings are crucial for understanding the evolutionary dynamics of IBV and developing more effective prevention and control strategies.

## Introduction

In chickens of all ages, infectious bronchitis (IB) is a highly contagious disease caused by the infectious bronchitis virus (IBV) (1, 2). IB inflicts significant economic losses on the poultry industry, resulting in lower egg production, higher feed conversion rates, and the scrapping of carcasses in slaughterhouses (3–5). These losses are particularly pronounced in the presence of nephropathogenic IBV strains or secondary infections (6). Vaccines are commonly employed to mitigate the economic damage caused by field strains of IBV (7, 8). However, IBV exhibits extensive antigenic and genetic diversity, continually giving rise to new genotypes, lineages, serotypes, and variants (3, 9–12). As such, managing and preventing IBV involves complex and difficult activities.

IBV is classified under the genus *Gammacoronavirus*, the family *Coronavirida* (13, 14). The IBV genome comprises a single-stranded positive-sense RNA, measuring approximately 27.6 kb in length. It encompasses at least 10 open reading frames (ORFs) (1). Among these ORFs, four encode the structural proteins spike (S), small envelope (E), membrane (M), and nucleocapsid (N) (1). The S protein is particularly important because of its virus-neutralizing epitope (15). The S glycoprotein undergoes cleavage, resulting in the formation of the S1 and S2 subunits. S1 is responsible for initiating IBV attachment to host cells, while S2 contributes to the viral fusion activity of IBV (16). The S2 subunit aids in identifying cellular tropism, and the phylogenetic study of the S1 gene permits the classification of IBV genotypes GI-GVII (9–12, 17–19). Moreover, one aspect of the S protein that remains largely unexplored is the role of its glycans. The glycosylation of S proteins in IBV plays a substantial role in determining multiple aspects of viral infection, including antigenicity, infectivity, and receptor binding (20–22). Consequently, it is imperative to focus on the potential alterations in glycosylation sites, particularly those of the S1 subunit, given their potential implications for the viral pathogenesis and immune evasion strategies.

Since its initial discovery, the GI-19 strains of IBV have become the main lineage within Chinese chicken flocks. Subsequently, this lineage has been observed in various countries and regions (23–26). Specifically, in China, the GI-19 strains have become the most commonly isolated IBV strains, with a prevalence that increased from 11.7% in 1994 to nearly 70% in 2016 (27, 28). The most effective method for preventing and managing IBV infection is still vaccination; however, the ongoing mutation and recombination of the IBV *S1* gene results in the emergence of variants against which protection by current vaccines is limited (29, 30). Therefore, long-term and continual monitoring of the pathogenicity, antigenicity, and molecular characteristics of IBV is crucial. This thorough investigation will advance our knowledge of the evolutionary dynamics of IBV in China, providing insightful guidance on how to address the IBV pandemic effectively.

The main aim of this study was to detect the major IBV genotype variants circulating in poultry flocks in East China and to monitor the possible emergence of any new IBV genotype. The present report describes the isolation and investigation of a highly pathogenic IBV strain (CK/CH/WJ215) belonging to the GI-19 lineage. The genetic and pathogenic characteristics and the tissue tropism of the isolate CK/CH/WJ215 isolate were investigated.

## Materials and methods

### Virus isolation

In May 2021, renal tissue samples were collected from a diseased commercial chicken flock in Jiangsu Province, China. The flock had received a coarse spray immunization with the H120 vaccine at 7 d of age. When the chickens reached 15 d of age, a small percentage of them exhibited extremely weak clinical symptoms. Subsequently, the broilers experienced sudden death between 25 and 32 d of age, with the majority of deaths occurring during this time frame. The morbidity rate was as high as 95%, while the mortality rate reached 65%. To identify the cause of the disease, reverse transcription-polymerase chain reaction (RT-PCR) was performed on pooled kidney samples taken from three sick broilers. The results of the RT-PCR test indicated the absence of avian influenza virus (AIV) subtypes H5 or H9, as well as the Newcastle disease virus (NDV). However, IBV was detected in the samples via RT-PCR test. According to the previously published protocol, the pooled kidneys were crushed with liquid nitrogen in an attempt to isolate the virus by inoculating it into the allantoic cavity of 10-d-old specific-pathogen-free (SPF) eggs (31). The kidney homogenate was subjected to blind passage 5 times, with 5 eggs involved in each passage. On the 5th d postinoculation, allantoic fluid samples were collected from all 5 inoculated eggs and subsequently assessed for the presence of Newcastle disease virus (NDV) and avian influenza virus (AIV) using hemagglutination (HA) activity testing. Additionally, the infectious bronchitis virus (IBV) was detected via RT-PCR. Furthermore, embryo lesions were investigated.

### RNA extraction and sequencing

RNA was extracted from allantoic fluid from the 5th passage for determination of the viral S1 nucleotide sequence. Briefly, RNA was extracted from 200 μL of allantoic fluid using an AxyPrep RNA Extraction Kit (AxyGen Bio, Inc., USA). The RNA was then reverse transcribed at 55°C for 45 min and 85°C for 5 min using the Goldenstar RT6 cDNA Synthesis Kit (Tsingke Biotech, China). For PCR amplification, the reaction mixture included 13 μL of Taq Plus Mix (Vazyme, China), 1 μL of each S1 primer (10 μM) (S1 Oligo5’: 5’-TGAAAACTGAACAAAAGAC-3′, S1 Oligo3’:5’-CATAACTAACATAAGGGCAA-3′) (32, 33), 2 μL of cDNA, and 8 μL of ddH_2_O. The thermocycling conditions were as follows: denaturation at 94°C for 3 min, followed by 35 cycles of 94°C for 30 s, 50°C for 30 s, and 72°C for 120 s, with a final extension step at 72°C for 5 min. The Phanta Super-Fidelity DNA polymerase (Vazyme, China) was used for amplification. The resulting PCR product had an approximate length of 1700 bp. Subsequently, DNA fragments were purified and ligated into a pGEM-T vector (Promega, China). The plasmid was transformed into DH5α competent cells, and 5 independent clones were sequenced by Tsingke Biotechnology (Nanjing, China).

### Phylogenetic analysis, glycosylation analysis, and recombination analysis

The *S1* gene sequence was BLAST searched using the National Center for Biotechnology Information (NCBI) database and then compared with representative sequences of IBV strains from different genotypes, as previously described (19), using MEGA version 11.0. The Tamura3-parameter model and the neighbor-joining technique were used, with bootstrap values set at 1,000.

The analysis of glycosylation sites on the S1 protein was performed using NetNGlyc – 1.0. Recombination analysis of the IBV S1 sequence was conducted using the RDP4 software. For recombination event detection, seven methods, namely, RDP, GENECONV, MaxChi, Chimera, BootScan, SiScan, and Phylpro, were employed. During the recombination analysis, the step sizes for the BootScan and SiScan detection methods were set at 50 and 20, respectively. The detection methods of RDP, BootScan, MaxChi, Chimera, and SiScan were configured with window sizes of 60, 200, 120, 120, and 500, respectively. The maximum *p* value threshold for the recombination analysis method was set at 1 × 10^−12^ to determine the reliability of the detected recombination events.

### Assessment of pathogenicity

The SPF chickens had unlimited access to food and water and were kept in separate isolators. Thirty-six 7-d-old SPF chickens were assigned numbers and randomly divided into two groups of 18 chickens each. In the infected group, the chickens were inoculated intranasally and intraocularly with 10^6^ 50% embryo infectious dose (EID_50_) of CK/CH/WJ215, while in the control group, the chickens were inoculated with SPF allantoic fluid. The body weights of all chickens were measured weekly following inoculation. Serum samples were collected from the remaining birds in the two groups at 3, 7, 14, 21, and 28 dpi for antibody detection using pELISA, as established previously (34). Three birds from each group were randomly marked and humanely killed on day 5 postinoculation, and trachea, lungs, kidneys, and bursa of Fabricius tissues were subjected to hematoxylin and eosin (H&E) staining (35). At dpi 3, 7, 14, 21, and 28 dpi, throat and cloacal swabs were collected to quantify viral loads through real-time quantitative polymerase chain reaction (RT-qPCR). A validated primer pair (IBV-F: GATGGTATAGTGTGGGTTGCT; IBV-R: GATGGTATAGTGTGGGTTGCT) was used to prepare a positive plasmid for the absolute quantification method as described in previous studies (36). The specific primers (qIBV-F: GGCAGAGTAGGCCAAGGTTT; qIBV-R: GCTAACCCACCATCCGCTAA) were designed using Premier 5 software and procured from Tsingke Biotechnology (Nanjing, China).

### Histopathology and immunohistochemistry

Tissues from the trachea, lung, kidney, spleen, and bursa were collected, fixed with 4% paraformaldehyde, and embedded in paraffin. The materials were then microscopically examined to evaluate alterations after being stained with H&E (35). Immunohistochemistry (IHC) was employed to determine the presence of IBV-specific antigens in the kidney, lung, and bursa samples, following established methods. Briefly, the tissue samples were fixed for 48 h, embedded in paraffin wax, sectioned into 5 μm slices, and stained with H&E. The IBV antigen was detection through IHC staining using an IBV N protein-specific monoclonal antibody.

### Statistical analysis

The data were analyzed utilizing GraphPad software. The body weights were analyzed using the Kruskal-Wallis test. The viral loads were analyzed by one-way ANOVA. Statistical significance was defined as a *p* value less than 0.05, while high significance was defined as a *p* value less than 0.01.

## Results

### Isolation and phylogenetic analysis of CK/CH/WJ215

Th allantoic fluid and pooled kidney samples were tested using RT-PCR and HA, and the results were negative for NDV and AIVs. However, RT-PCR analysis revealed a positive result for IBV in the samples. Subsequently, an IBV strain named CK/CH/WJ215 was successfully isolated from 10-d-old SPF chicken embryos.

**Figure 1:**
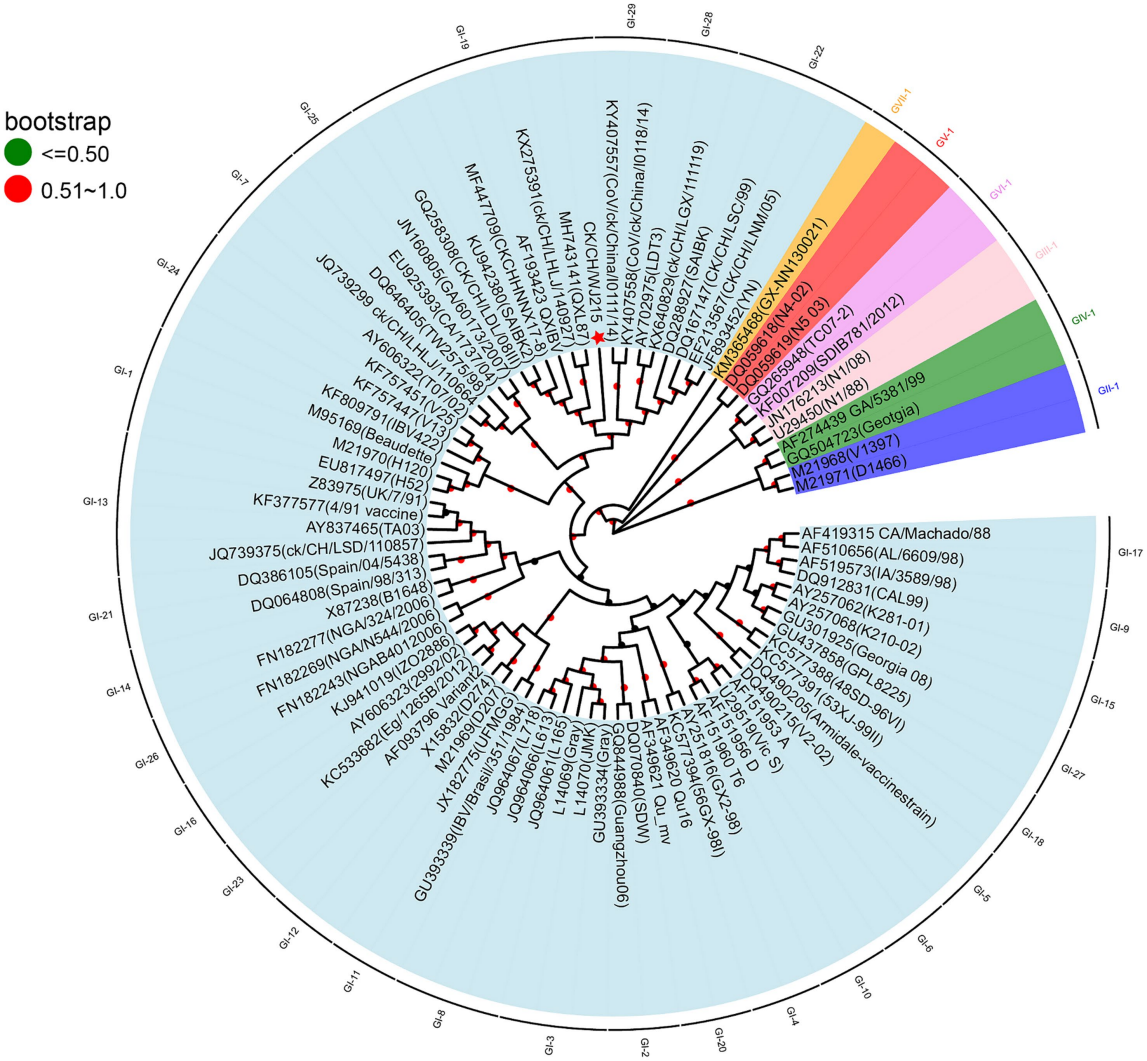
Phylogenetic analyses of the S1 gene of the isolate CK/CH/WJ215. Nucleotide sequence alignments of the S1 gene of CK/CH/WJ215 (represented by a red star) and 82 reference strains were used to construct phylogenetic trees. The MEGA 11.0 software was used for this analysis, and the maximum likelihood method was applied with 1,000 bootstrap replicates. The phylogenetic analysis included 82 reference strains, representing seven different genotypes (GI-GVII) and 36 lineages. The S1 gene was specifically chosen for this analysis.

To better understand the biological and ecological aspects of the recently discovered IBV strain, we conducted a sequencing analysis of the *S1* gene of CK/CH/WJ215. The resulting sequence was then submitted to the NCBI with the accession number PP212853. Subsequent BLAST analysis indicated that the isolate closely resembled the GI-19 genotype strain 42HLJ-98I (91.2%), which was isolated from broilers in Guangdong Province. According to a sequence homology study based on the S1 gene, CK/CH/WJ/215 shares low identity (75.7, 78.6, and 77.5%) with the three vaccine strains, H120, LDT3-A, and 4/91. To comprehensively investigate the genetic relationships between CK/CH/WJ215 and other IBV strains, a phylogenetic tree was constructed using the S1 gene sequences of 84 reference strains. These reference strains included 7 genotypes and 36 lineages (10, 19). The phylogenetic tree (Figure 1) that was generated indicated that the GI-19 genotype and CK/CH/WJ215 belonged to the same branch.

### CK/CH/WJ215 shows distinct glycosylation sites within its S1 protein

**Figure 2:**
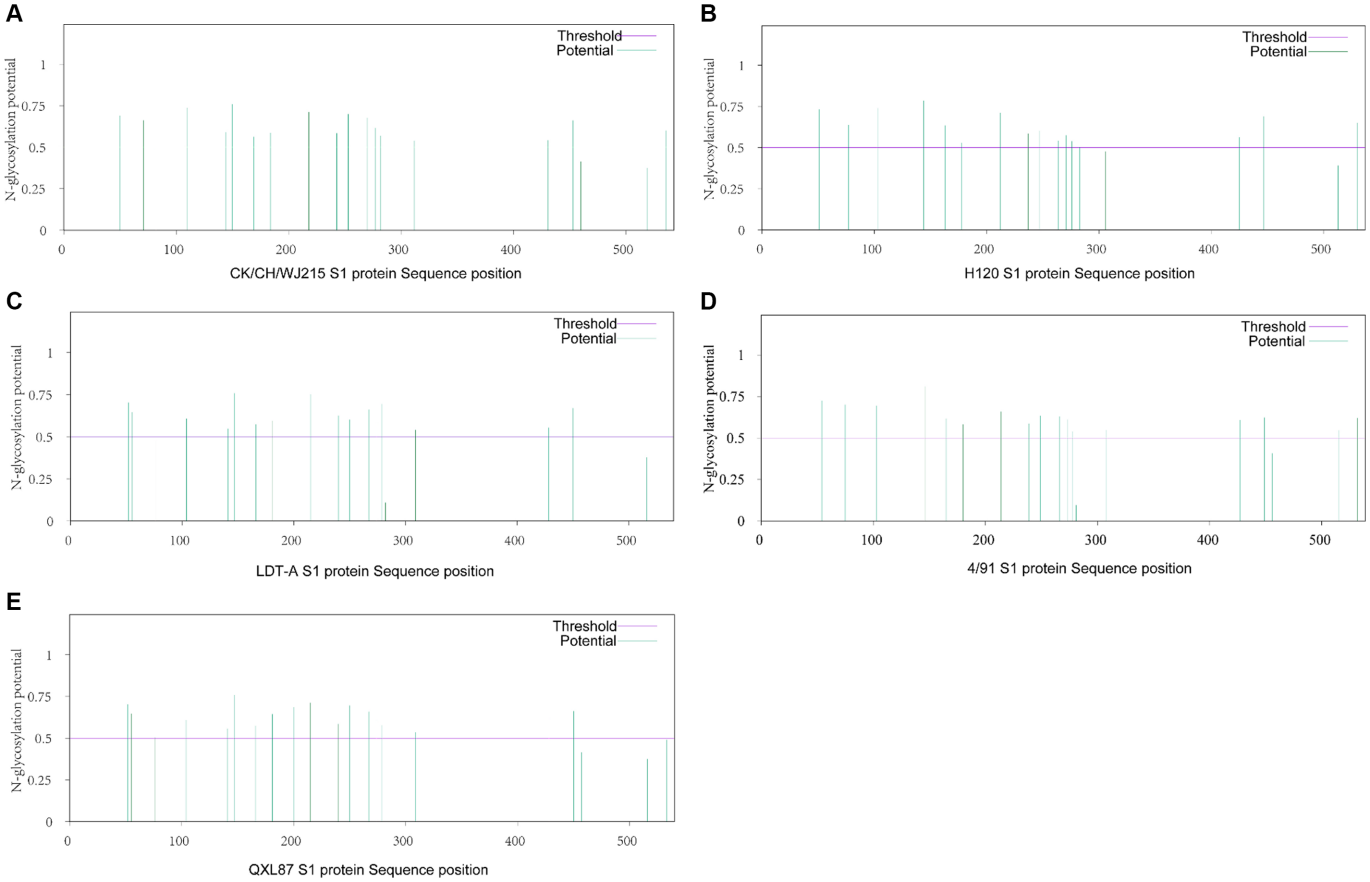
Prediction of potential N-glycosylation sites in the IBV S1 protein. **(A)** Prediction of potential N-glycosylation sites of the CK/CH/WJ215 S1 protein. **(B)** Prediction of potential N-glycosylation sites of the H120 S1 protein. **(C)** Prediction of potential N-glycosylation sites of the LDT-A S1 protein. **(D)** Prediction of potential N-glycosylation sites of the 4/91 S1 protein. **(E)** Prediction of potential N-glycosylation sites in the QXL87 S1 protein.

Further analysis of the potential glycosylation sites of the S1 protein in the IBV strains revealed that the CK/CH/WJ215 isolate exhibited 17 potential potential N-glycosylation sites, similar to those in the QXL187 and 4/91 vaccine strains, whereas the S1 proteins of the H120 and LDT-A strains exhibited 16 potential sites. This comparative analysis highlights the variation in glycosylation profiles among different IBV strains (Figure 2). Comparison with commonly used vaccine strains such as H120, 4/91, LDT-A, QXL87 showed variations in 10 potential glycosylation sites (Figure 2). Specifically, compared to those of the H120 and 4/91 vaccine strains, a potential glycosylation site appeared at position 138, and variations were observed at positions 103, 247, and 530, resulting in a change from phenylalanine (F) at position 104 to tyrosine (Y), from serine (S) at positions 248 and 249 to threonine (T), and from glycine (G) and threonine (T) at positions 531 and 532 to serine (S) (Table 1). Additionally, variations were also observed at positions 51, 77, 163, and 276 in the CK/CH/WJ215 strain compared to the H120 strain, altering the glycosylation activity at these sites. Compared to those in the LDT-A and QXL87 vaccine strains, a glycosylation site appeared at position 530, and mutations occurred at positions 103 and 247, leading to a change from phenylalanine (F) at position 104 to tyrosine (Y) and from serine (S) and threonine (T) at positions 248 and 249 to threonine (T) (Table 1). Furthermore, a comparison with the H120 strain revealed the absence of a potential glycosylation site at position 283 of the CK/CH/WJ215 S1 protein.

**Table 1:** Differences in potential glycosylation sites.

Strain ID	Glycosylation site									
	51	77	103	138	163	178	247	276	283	530
H120	NISS	NASS	NFSD	/	NLTS	NETT	NSSL	NETG	NPSG	NGTR
4/91	/	NISA(75)	NFSD(103)	/	NSTS(165)	NETT(180)	NSSL(249)	NVSN(278)	/	NGTR(532)
LDT-A	NSTN(52)	NYTN(55)	NFSE(104)	NGSL(141)	NFTS(166)	NITT(181)	NSSL(250)	/	/	/
QXL87	NSTN(52)	NQSA(76)	NFSE(104)	NGTL(141)	NLTS(166)	NKTT(181)	NSTL(250)	NVSN(279)	/	/
CK/CH/WJ215	NSTS(50)	NQSA(82)	NYSD(110)	NGSL(144)	NFTS(169)	NETT(184)	NTTL(253)	NVSN(282)	/	NSSR(533)

“/” represents no potential glycosylation site. The numbers represent the position of the H120 S1 protein amino acid. The numbers in brackets represent the position of the amino acid S1 for each strain.

### CK/CH/WJ215 was highly pathogenic to the SPF chickens

**Figure 3:**
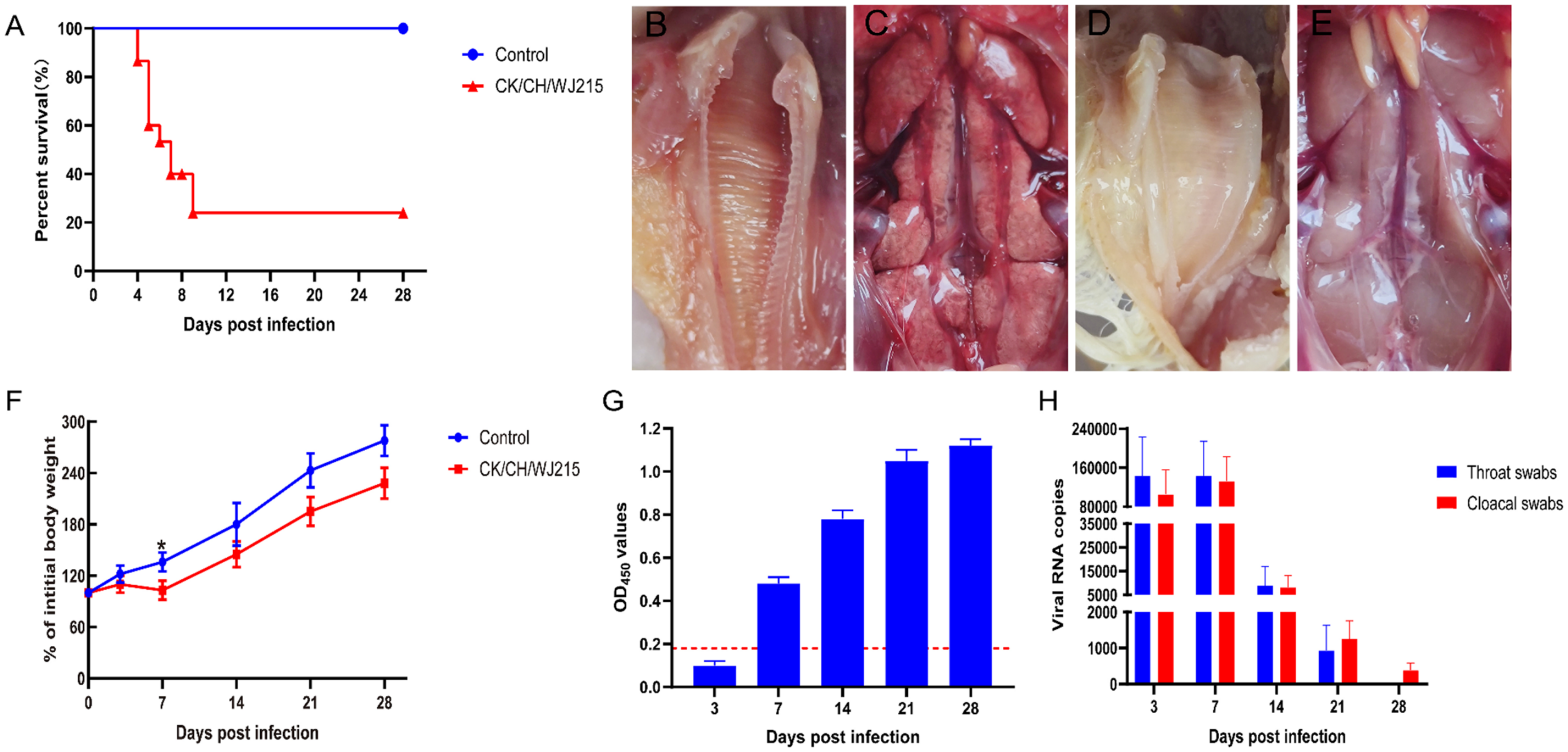
Pathogenicity of the CK/CH/WJ215 isolates in chickens. **(A)** Survival percentage of chickens infected with the CK/CH/WJ215 strain. **(B–E)** The trachea and kidneys of the control and infected groups, respectively. **(F)** The percentage of body weight gain of chickens infected with the CK/CH/WJ215 strain. **(G)** The antibody level of chickens infected with the CK/CH/WJ215 strain. **(H)** Viral loads in throat swabs and cloacal swabs from chickens infected with the CK/CH/WJ215 strain. The error bars indicate standard deviations.

When the CK/CH/WJ215 strain was inoculated into 7-d-old SPF chickens, approximately one-third of the animals developed clinical signs at 2 dpi, including depression, disheveled feathers, nasal discharge, tracheal rales, white watery feces, and sticky fluid in the eyes and nose. Mortality began at 4 dpi, with a total of 11 deaths by the 9 dpi. Up to 5 dpi, all surviving chickens displayed symptoms of depression and tracheal rales. The mortality rate was 73.3% (11/15), and the deceased SPF chickens exhibited severe dehydration (Figure 3A). Inspection during necropsy revealed that the kidney had a significant buildup of urate deposits and was pale in color and swollen. Additionally, slight congestion bleeding was detected in the trachea of infected group chickens (Figure 3C), whereas typical urate deposition was detected in the kidney of infected group chickens (Figure 3B). No pathology was detected in the trachea or kidney of the control group chickens (Figures 3D,E). Moreover, at 7 dpi, the body weight of IBV-infected chickens was significantly less than that of control chickens. However, no significant difference was observed between the infected and control groups at 14, 21, and 28 dpi according to pairwise comparisons (Figure 3F). Interestingly, IBV antibodies were detected after 7 d of infection, with antibody levels gradually increasing over time (Figure 3G). Furthermore, the viral genome copy numbers in the throat and cloacal swabs of the infected group were consistently greater than 10^5^ at 3 dpi and 7 dpi. Notably, IBV RNA was still detected in cloacal swabs of the infected group at 28 dpi (Figure 3H).

### CK/CH/WJ215 exhibited broad tissue tropism in the infected SPF chickens

**Figure 4:**
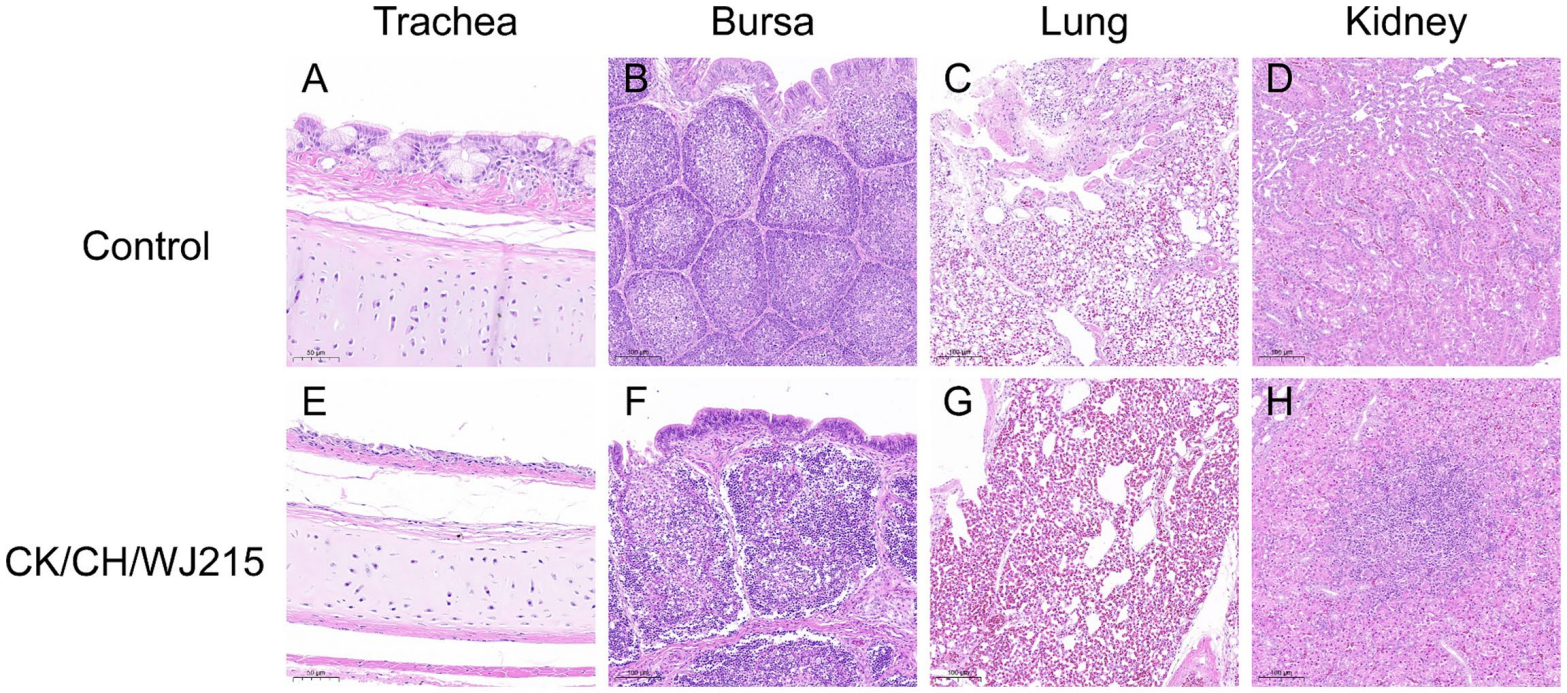
Histopathological alterations were identified in several tissues of the chickens. Tissues **(A–D)** correspond to the trachea, bursa of Fabricius, lung, and kidneys of the negative control group chickens at 5 dpi, respectively. On the other hand, tissues **(E–H)** demonstrate histopathological lesions specifically in the trachea, bursa of Fabricius, lung, and kidneys of the chickens in the infected group at 5 dpi.

**Figure 5:**
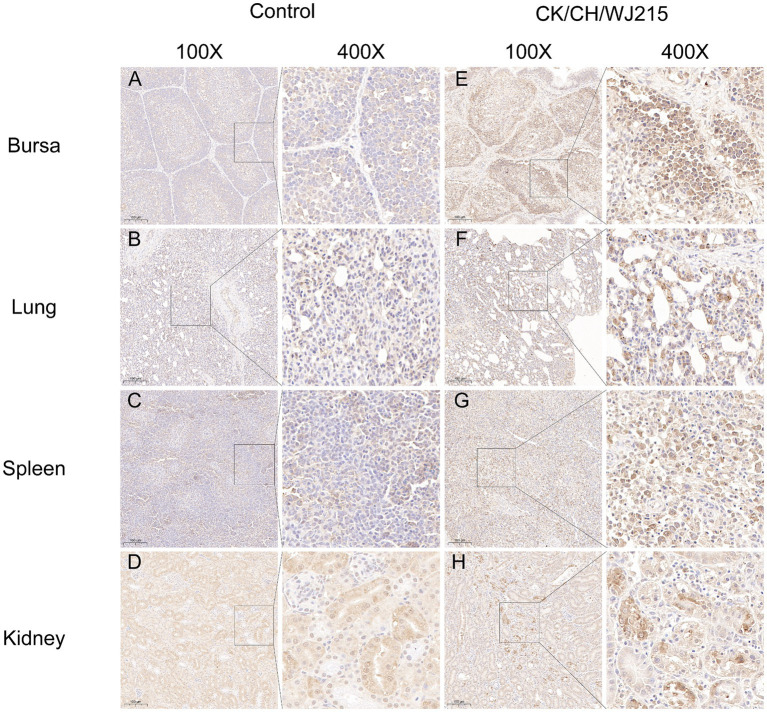
The IBV antigens were evaluated in both the infected group and the control group by utilizing immunohistochemistry. Tissues from deceased chicks were collected at 5 dpi. Immunohistochemical staining was performed, and a brown color signal indicated the presence of virus antigens in the trachea **(E)**, lung **(F)**, spleen **(G)**, and kidneys **(H)**, using a monoclonal antibody specific for the IBV N protein. Tissues **(A–D)** belonged to the negative control group chickens at 5 dpi. The IBV N protein was detected by an IBV N protein monoclonal antibody.

In the infected cohort, the tracheal mucosa exhibited a prominent loss of epithelial cells, characterized by widespread shedding and necrosis, in contrast to the control group, where the tracheal epithelium maintained its integrity, displaying intact ciliated cells (Figures 4A,E). The microscopic examination of the bursa of Fabricius, lungs, and kidneys from the control chickens revealed no histopathological changes indicative of IBV infection (Figures 4B–D). Conversely, the bursa of Fabricius in the infected group demonstrated a significant depletion of lymphocytes when compared to that in the uninfected controls, suggesting immunocompromise (Figure 4F). The pulmonary parenchyma of the infected birds exhibited pronounced hemorrhage and vascular congestion, indicative of a severe inflammatory response (Figure 4G). Moreover, the renal tissue of the infected group exhibited manifested an aggressive infiltration of inflammatory cells, consistent with a proactive host immune response to IBV-induced renal pathology (Figure 4H). As illustrated in Figure 5, the control group did not exhibit any discernible viral antigens. In contrast, the IBV-infected group showed clear evidence of viral N protein antigens in the cytoplasm of the cells of different organs, such as the bursa, lung, spleen, and kidney (Figure 5).

## Discussion

IBV has spread rapidly throughout China as a result of the country’s growing chicken farming industry. Currently, the epidemiology of infectious bronchitis is complex in China, and despite the widespread use of IBV vaccines, outbreaks of IBV infection are still frequent (37, 38). IB primarily affects the respiratory, renal, and reproductive systems, with an incidence rate of up to 100%. However, the case fatality rate varies greatly, ranging from 0 to 82% (39). High mortality rates are observed in patients with renal lesions, leading to significant economic losses in China’s poultry industry (27, 38). Elucidating the genetic and biological properties of IBV strains in chickens, such as tissue tropism, pathogenicity, and antigenicity, can aid in understanding the evolution of IBV. In this study, an IBV strain was isolated and identified from diseased chicken samples obtained from a poultry farm in Jiangsu. After 5 successive blind passages in SPF chicken embryos, the strain elicited typical symptoms of stunted development and thickening of the urinary bladder membrane in dwarf embryos, leading to severe chick embryo mortality, indicating that the isolated strain CK/CH/WJ215 adheres to the pathogenic characteristics of IBV infection in chicken embryos. Animal pathogenicity tests revealed that 7-d-old SPF chickens infected with the CK/CH/WJ215 strain exhibited typical clinical symptoms, including depression, disheveled feathers, tracheal rales, white watery droppings, and mortality. Necropsy of the deceased chickens revealed typical mosaic kidneys with extensive urate deposition in the ureters and cloaca. The isolated strain is a relatively typical IBV strain with considerable pathogenicity, in line with other investigations, as evidenced by its 100% morbidity and 73.3% mortality.

Currently, the predominant IBV genotype in China is GI-19, which was initially discovered in chickens showing proventriculitis in that nation in 1996 (40). The GI-19 genotype of IBV has been reported worldwide, including in Europe, Asia, Africa, and the Middle East (23, 26, 41, 42). It has been detected in cases of IB-related egg production abnormalities, false layer syndrome, and nephritis, posing a significant threat to the global poultry industry. Yuan et al. conducted an epidemiological survey of IB in southern China from 2021 to 2022 and found that the GI-19 genotype still occupied a major position among the isolated viruses during this period. In recent years, the proportion of IBV strains with the GI-19 genotype has been increasing, and this genotype has become dominant in some regions (27). Researchers have regularly obtained IBV strains with the GI-19 genotype from immunized chicken flocks, and the majority of these strains exhibit genetic recombination events (26, 37, 43–45). The CK/CH/WJ215 strain isolated in this study was isolated from a flock of chickens immunized with the H120 vaccine strain. Identity analysis revealed that the S1 gene of the CK/CH/WJ215 strain has a low identity (approximately 75%) to vaccine strains such as H120, 4/91, and LDT-A, indicating that the H120 vaccine strain used in some domestic chicken farms may not effectively prevent the occurrence of similar GI-19 genotype IBV strains (46). It is necessary to conduct epidemiological monitoring of IBV and select appropriate vaccines and immunization procedures based on the local situation. Gene recombination of the *S1* gene is an important factor in IBV variation, determining tissue tropism and pathogenicity (18, 20, 37). The CK/CH/WJ215 strain, which belongs to the GI-19 genotype, has a close affinity to the 42HLJ-98I strain that was recovered from Guangdong Province, according to a genetic evolution study, suggesting that the IBV epidemic scenario is still complex.

The initial stage of viral infection is caused by the attachment of the IBV S1 protein to host cell receptors. The only known IBV receptors at this time are alpha-2,3-sialylated glycoproteins (47, 48). Posttranslational modifications such as glycosylation may aid in protein folding and play important roles in viral receptor binding and virus-cell and cell–cell fusion (21, 22). Bouwman et al. demonstrated that N-glycosylation sites in the receptor-binding domain of the IBV S1 protein determine receptor binding specificity (49). N-glycosylation at different positions may have different impacts on S protein-mediated fusion, virus infectivity, and replication. Zheng et al. reported that N-glycosylation mutations at positions 144 and 247 significantly reduced the cell–cell fusion ability of IBV, while mutations at position 276 did not induce cell–cell fusion. Strains with mutations at positions 51/77, 144, and 163 exhibited slower growth rates than did the wild-type virus, indicating the importance of glycosylation sites 51, 77, 144, 163, and 247 in IBV-induced cell–cell fusion and virus replication/infection (22). *In silico* docking studies and glycosylation analysis revealed a potential ligand-receptor site that is flanked by 51, 77, 103, and 144 glycosylation sites that dramatically impact ligand binding. The CK/CH/WJ215 strain possesses these glycosylation sites, albeit with altered glycosylation potential compared to that of vaccine strains. Further research is essential to investigate whether these modifications influence ligand binding affinity. In addition, the CK/CH/WJ215 strain displayed variant glycosylation at positions 138 and 530 in comparison to the H120, 4/91, LDT-A, and QXL87 vaccine strains. These differences indicate the potential benefits of the CK/CH/WJ215 strain in receptor binding and triggering cell-to-cell membrane fusion.

One important way that IBV spreads is through the constant viral shedding that infected hens release into the environment. In this study, RT-PCR was employed to monitor the viral shedding in tracheal and cloacal swabs. The results indicated that at 28 dpi, the detection rate of viral shedding in cloacal samples remained at 100%. These results show that the isolated strains infected the chicken population and caused persistent shedding and high infectivity. Given the prolonged shedding by infected chickens, diseased and carrier birds serve as the primary sources of viral contamination, leading to the establishment of a sustained infection. Consequently, the control and prevention of IBV rely heavily on addressing this persistence, which represents a major challenge.

## Conclusion

An isolate of the GI-19 genotype IBV (CK/CH/WJ215) from Jiangsu Province was used in this investigation. This isolated strain typically exhibits renal pathogenic characteristics and shows low homology with commonly used vaccine strains. Further analysis revealed variations in ten N-glycosylation sites between different genotype vaccine strains, such as H120, 4/91, LDT-A, and QXL87. These traits could be linked to its high pathogenicity in vaccinated chickens.

## Data availability statement

The data presented in this study can be found in online repositories. The names of the repository/ repositories and accession number(s) can be found at: https://www.ncbi.nlm.nih.gov/genbank/, PP212853.1.

## Ethics statement

The animal study was approved by Experimental Animal Ethics Committee, Jiangsu Academy of Agricultural Sciences. The study was conducted in accordance with the local legislation and institutional requirements.

## Author contributions

QW: Funding acquisition, Methodology, Writing – review & editing. MX: Formal analysis, Investigation, Writing – original draft. DW: Investigation, Writing – original draft. XZ: Data curation, Writing – original draft. DL: Validation, Writing – original draft. MM: Project administration, Writing – review & editing.
